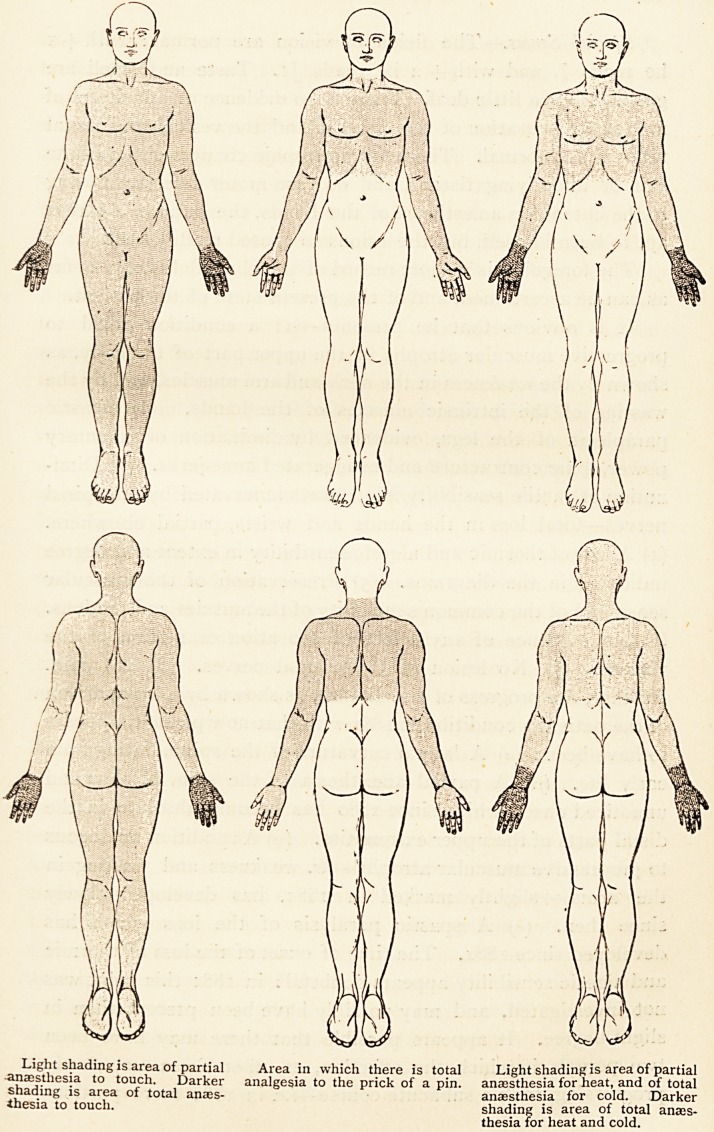# A Case of Syringomyelia
1A Paper read at a meeting of the Bristol Medico-Chirurgical Society, March, 1894.


**Published:** 1894-06

**Authors:** F. H. Edgeworth

**Affiliations:** Assistant-Physician to the Bristol Royal Infirmary


### A CASE OF SYRINGOMYELIA.[1]

BY

F. H. Edgeworth, M.B., B.A. Cantab., B.Sc. Lond.,

*Assistant-Physician to the Bristol Royal Infirmary.*

---

The patient whom I have shown to-night was admitted to the Royal Infirmary on February 20th, 1894, under my care, complaining of weakness and of inability of feeling objects with his hands. He is aged 57, never married, by occupation a messenger and potman. His father died at the age of 57 of gout; his mother, aged 37, of an unknown cause. He has four sisters living and healthy; there are no brothers living, two died (one in infancy) of unknown causes.

His physical history is as follows: When aged 7 years he received an injury to the right hand, deforming the thumb; in later childhood a lateral curvature of the spine slowly appeared. When aged 27 he had typhoid fever (under Dr. Beddoe, at the Royal Infirmary). In 1881 he was admitted to the Royal Infirmary, under the care of Dr. Shaw. The notes then taken of his condition are valuable, as they show that some of the symptoms which he now presents existed then, and that others have developed since that time. I need then make no apology for quoting them nearly in full: "Eight days before admission the patient found, somewhat suddenly, that he could not raise his arms above the shoulders; there was no fit, or loss of consciousness, or pain in the arms at the time of onset. The arms got worse up to the time of admission. There was no history of syphilis or lead-poisoning. On examination it was found that the temperature was normal, heart and lungs healthy. Marked lateral curvature of spine. No bladder or rectal trouble. Cannot put arms above head; movements of elbows and shoulders easily restrained, wrist movements slow and feeble, hardly any grasp in either hand. Leg movements good; both

[1] A Paper read at a meeting of the Bristol Medico-Chirurgical Society, March, 1894.

plantar reflexes present. Knee-jerks present, no rectus or ankle clonus. Cremasteric, abdominal, and epigastric reflexes present. No wasting of legs or arms. Neither deltoid reacts well to faradic current, all other muscles easily. Sensation rather dulled over both arms and over trunk from trunk to groin. Eyes normal." The patient after a time went out, neither better nor worse than when he entered the Infirmary. He now says that a fortnight before admission, on February 20th last, he found he could not feel objects with his hands, and that this loss of sensibility came on rather suddenly.

On examination, it is found that the patient is a little man, looking older than his years, with arcus senilis. The heart, lungs, abdomen, urine, are normal; appetite good, bowels open every other day, sleeps well. There is a marked lateral curvature of the spine to the right, involving the whole dorsal region, with slight compensatory curves above and below.

*Muscles.*—The pupils are equal in size, of medium diameter, contract well to light and accommodation, but do not dilate on irritation of the skin. The movements of the external ocular muscles are normal. The facial, glossal, palatal, pharyngeal and laryngeal muscles are normal. The head can be rotated well, but the lateral movements are feeble and slow. In the right arm the terminal phalanx of the thumb is anchylosed to the proximal one. All the fingers, in an increasing degree towards the ulnar side, are moderately flexed at all joints: this flexion can be voluntarily increased at all joints so as to form a fist, but the fingers cannot be extended either voluntarily or by a bystander, probably due to shortening of the long flexor tendons. The grasp of the hand is exceedingly weak. There is wasting of the interossei muscles and of the thenar and hypothenar eminences. The fingers cannot be abducted or adducted; but the thumb movements are executed, though feebly. In the left arm there is slight ulnar deflection of the little finger, probably due to rheumatoid arthritis. Abduction and adduction of the fingers are possible. The thenar and hypothenar eminences are flat, but all movements of the thumb are performed. All movements of fingers, hand, wrist, forearm and arm are executed with exception of that of abduction at the shoulder-joint, which cannot be completely performed. There is no obvious wasting of the muscles of the left forearm and arm, but all the movements described above are very feebly performed. In the legs the patient says his power is feeble, and that his knees feel weak, especially on going up stairs. On asking the patient to walk he presents an obviously spastic gait. The muscles are moderately developed: there is no wasting, and all movements are performed on request, though feebly. There is slight tonic contracture in the adductor and hamstring muscles of the thigh and calf muscles in both legs. The trunk movements are all possible, but are feebly performed. On electrical examination, it is found that there is no reaction of degeneration, but that there is a marked diminution in response to both constant and interrupted currents in the thenar and hypothenar muscles of both hands and in the interossei of the left hand, whilst there is no contraction of the interossei muscles of the right hand on the application of either current. There is also slight diminution to both currents in all the muscles of both arms.

*Reflexes.*—The scapular, gluteal, epigastric, abdominal and cremasteric reflexes are absent; the plantar reflexes are both present and somewhat active. Both knee-jerks are exaggerated, but neither rectus nor ankle clonus can be elicited on either side. The triceps and biceps jerk are absent in both arms.

*Sensation.*—The loss of tactile sensation is as represented in the diagram. Sensibility to heat, cold, and painful impressions is lost in the area indicated in the diagram: it is somewhat noteworthy that the area is almost identically the same for these three forms of sensibility. In testing the sensibility to heat, it is noticeable that in the skin-area of loss, the hot object (a test-tube filled with hot water), though it is not felt, produces a decided flush on the part to which it is applied, probably due to a local action on the skin capillaries. The muscular sense is everywhere normal, as is also the common sensibility of the muscles—*e.g.* in the lower part of the forearms, where there is absolute cutaneous anæsthesia; if a pin be thrust through the skin, a painful sensation is felt directly the point touches the muscles or tendons.

Light shading is area of partial anæsthesia to touch. Darker shading is area of total anæsthesia to touch.

Area in which there is total analgesia to the prick of a pin.

Light shading is area of partial anæsthesia for heat, and of total anæsthesia for cold. Darker shading is area of total anæsthesia for heat and cold.

*Special Senses.*—The fields of vision are normal: with + 1 he reads $\frac{5}{6}$, and with + 2 he reads J1. Taste and smell are good. He is a little deaf. There is no evidence of any defect of motion or sensation of the viscera, and the vesical and rectal reflexes are normal. There are no trophic changes either of the skin or underlying tissues, and no vaso-motor defects. Owing to the cutaneous anæsthesia of the hands, the patient is rather apt to burn himself, but the lesions so caused readily heal.

The foregoing is a short record of the clinical history, as far as can be ascertained, and of the present state of the patient.

It is obvious that he presents—(1) a condition allied to progressive muscular atrophy in the upper part of the body, as shown by the weakness in the neck and arm muscles, and by the wasting of the intrinsic muscles of the hands. (2) Spastic paraplegia of the legs, evidenced by diminution of voluntary power, tonic contracture and exaggerated knee-jerks. (3) Diminution of tactile sensibility in all parts innervated by the spinal nerves—total loss in the hands and wrists, partial elsewhere. (4) A loss of thermic and algesic sensibility in extent and degree indicated in the diagrams. (5) Preservation of the muscular sense and of the common sensibility of the muscles and tendons. (6) No evidence of any defect of sensation or motion of the viscera. (7) No lesion of the cranial nerves. (8) No pain. Further, the progress of the disease, as shown by a comparison of the patient's condition in 1881 with that now present, appears to have been: (*a*) A lateral curvature of the spine, dating from early life. (*β*) A partial anæsthesia of the skin, of slow and unnoticed onset, which since 1880 has become absolute in the distal parts of the upper extremities. (*γ*) A condition analogous to progressive muscular atrophy—*i.e.* weakness and wasting in the arms — slightly marked in 1881, has developed slowly since then. (*δ*) A spastic paralysis of the legs which has developed since 1881. The time of onset of the loss of thermic and algesic sensibility appears doubtful: in 1881 this point was not investigated, and may possibly have been present then in slight degree. It appears possible that there may have been two periods in which the affection, at other times very slowly progressing, took a subacute course—*i.e.* in 1881 more particularly in respect to the loss of power in the arms, and in 1894 as regards the loss of tactile sensibility in the arms.

The diagnosis arrived at from a consideration of the above points is that of syringomyelia. It is obvious that hysteria, myelitis, cervical pachymeningitis, spinal tumour and peripheral neuritis (which are the affections most liable to be confused with the above) can be excluded. The typical symptoms of this condition are—(1) Progressive muscular atrophy in the arms, (2) spastic paraplegia of the legs; (3) loss of skin sensibility of wider extent and earlier onset than the muscular wasting, generally affecting only thermic and algesic sensibility.

This case, then, is atypical, in that there is a loss of tactile sensibility, of distribution and degree described above. With reference to this point, it is to be remarked that this symptom has been marked in cases in which the diagnosis of syringomyelia has been verified. Hence, it does not invalidate the diagnosis.

Again, there are no trophic lesions—the case is one of simple syringomyelia, and not one of Morvan's disease, which is apparently syringomyelia *plus* peripheral neuritis, and which is characterised clinically by the addition of tactile anæsthesia and trophic lesions (the most typical of the latter being painless indolent whitlows of the fingers) to those of syringomyelia.

As the disease is somewhat rare (less than a hundred cases having been recorded), and as this is the first instance shown at this Society, I may perhaps add a short account of the pathology as far as it is known, to make the connotation of the term clearer. To do so, we must begin at the beginning. The spinal cord (and brain) is formed by the invagination of a median longitudinal strip of dorsal ectoderm, which separates off and then consists of an oval tube with cellular walls. The white matter—*i.e.* nerve-fibres—is formed on the exterior of this tube by outgrowths of cell processes; and the anterior fissure is due to lateral downgrowths of the grey matter, covered by the white, on either side of the median ventral line. The posterior fissure is formed in a different way: the central canal is obliterated from above downwards (*i.e.* back to front, if the embryo be placed upright) by a collapse of its walls, so that only the ventral portion persists as the small central canal of the cord, and the posterior fissure is due to absorption of the dorsal wall so formed from above downwards.[1] Now, syringomyelia is due primarily to a persistence of this long oval embryonic central canal, having walls composed of embryonic neuroglia tissue. This condition, sometimes called hydromyelia, causes no symptoms, and is found accidentally at autopsies.

The symptoms of syringomyelia are due to secondary processes, either to distension of the persistent embryonic canal or to overgrowth, with occasionally secondary breaking down of the neuroglia wall. The distension is usually most marked in the cervical and upper dorsal region, and correspondingly the symptoms as a rule begin and are most marked in this region. The distension, if extreme, will affect the anterior cornua causing atrophy of the corresponding muscles, and the pyramidal tracts causing spastic paraplegia of the legs; but these motor affections are always slighter in degree than, and secondary in time to, the sensory symptoms which are due to involvement of the posterior cornua and sensory tracts. The typical impairment is that of thermic and algesic sensibility. Why this should be is not known, nor is it known why tactile sensibility usually, and muscular and visceral sensibility always, are normal.

The main sensory tracts in the cord are generally held to be the posterior white columns, together with the dorsal and ventral cerebellar tracts of the antero-lateral white columns. Of these only the fibres of the posterior-median columns are directly continuous with those of the posterior roots; the grey matter of the cord intervenes between the fibres of the other tracts and the posterior roots. As regards function, there is a good deal of evidence that muscular sense-impulses pass up the posterior-median columns on the same side as they enter; whilst the evidence is conflicting for other kinds of sensory impulses. Clinical and pathological facts have generally been held to show that cutaneous (of heat, cold, pain and touch) impulses cross the

1 Some authorities hold that the posterior median fissure is formed not merely at the expense of, but partially from, the dorsal part of the large embryonic central canal.

cord soon after entry and run up on the other side, whilst recent experimental research points to the view that sensory impulses mainly pass up on the side of entry.

The evidence deduced from cases of syringomyelia does not shed any light on the problem of decussation in the cord; but it perhaps indicates that the grey matter, and hence those kinds of impulses which pass through it, is more affected by the enlarging central canal than are the posterior white columns.

## Figures and Tables

**Figure f1:**